# Distinguishing cognitive state with multifractal complexity of hippocampal interspike interval sequences

**DOI:** 10.3389/fnsys.2015.00130

**Published:** 2015-09-17

**Authors:** Dustin Fetterhoff, Robert A. Kraft, Roman A. Sandler, Ioan Opris, Cheryl A. Sexton, Vasilis Z. Marmarelis, Robert E. Hampson, Sam A. Deadwyler

**Affiliations:** ^1^Neuroscience Program, Wake Forest School of MedicineWinston-Salem, NC, USA; ^2^Department of Physiology and Pharmacology, Wake Forest School of MedicineWinston-Salem, NC, USA; ^3^Department of Biomedical Engineering, Wake Forest School of MedicineWinston-Salem, NC, USA; ^4^Department of Biomedical Engineering, University of Southern CaliforniaLos Angeles, CA, USA

**Keywords:** delayed nonmatch-to-sample, hippocampus, long-range temporal correlations, multifractal detrended fluctuation analysis, resting state, tetrahydrocannabinol (THC), working memory

## Abstract

Fractality, represented as self-similar repeating patterns, is ubiquitous in nature and the brain. Dynamic patterns of hippocampal spike trains are known to exhibit multifractal properties during working memory processing; however, it is unclear whether the multifractal properties inherent to hippocampal spike trains reflect active cognitive processing. To examine this possibility, hippocampal neuronal ensembles were recorded from rats before, during and after a spatial working memory task following administration of tetrahydrocannabinol (THC), a memory-impairing component of cannabis. Multifractal detrended fluctuation analysis was performed on hippocampal interspike interval sequences to determine characteristics of monofractal long-range temporal correlations (LRTCs), quantified by the Hurst exponent, and the degree/magnitude of multifractal complexity, quantified by the width of the singularity spectrum. Our results demonstrate that multifractal firing patterns of hippocampal spike trains are a marker of functional memory processing, as they are more complex during the working memory task and significantly reduced following administration of memory impairing THC doses. Conversely, LRTCs are largest during resting state recordings, therefore reflecting different information compared to multifractality. In order to deepen conceptual understanding of multifractal complexity and LRTCs, these measures were compared to classical methods using hippocampal frequency content and firing variability measures. These results showed that LRTCs, multifractality, and theta rhythm represent independent processes, while delta rhythm correlated with multifractality. Taken together, these results provide a novel perspective on memory function by demonstrating that the multifractal nature of spike trains reflects hippocampal microcircuit activity that can be used to detect and quantify cognitive, physiological, and pathological states.

## Introduction

By analyzing the mono- and multifractal properties of neural temporal dynamics, we may generate new insights concerning how the brain functions with implications for detection of cognitive, physiological, and pathological states. Such analyses have been used successfully to detect pathological conditions such as heart disease (Ivanov et al., 1999), Alzheimer's disease (Lahmiri and Boukadoum, 2013), Parkinson's disease (Zheng et al., 2005), and epilepsy (Serletis et al., 2012; Dutta et al., 2014). Additionally, multifractal analysis detects clear differences in neural activity between wakefulness and sleep stages using EEG signals (Weiss et al., 2009; Zorick and Mandelkern, 2013). Multifractal complexity of time series is believed to indicate functional connectivity because such complexity would provide the essential temporal dynamics to support information transfer via variable patterns of neural activity. Interactions across brain regions, detected as multifractal complexity, regularly fluctuate between task and rest conditions specifically in regions associated with the task (Ciuciu et al., 2012). In order to assess cognitive state detection abilities, a paradigm was implemented to examine how multifractal complexity is reflected by active (i.e., task-related) hippocampal microcircuit processing.

Temporal coding analyses attempt to derive information about brain function from the timing of action potentials generated by neuronal ensembles or from rhythmic neuronal oscillations, such as theta rhythm (Jones and Wilson, 2005). Some temporal coding analyses assign physiological function to activity within frequency bands (i.e., theta-phase precession or cross frequency coupling), but we propose that multifractal analysis can provide new insights into mechanisms of neurological temporal coding because it quantifies the structure of variability and the self-similar (fractal) nature of physiological systems. Scale-free dynamics are often associated with long-range temporal correlations (Linkenkaer-Hansen et al., 2001; Ciuciu et al., 2012) and quantified by the monofractal Hurst exponent. However, a single exponent does not capture the complexity of many physiological signals, supporting the use of a spectrum of scale invariant exponents that describe the multiple, co-existing fractal patterns (Dixon et al., 2012). Memory is commonly believed to occur through repetitive neuronal sequences (Hampson et al., 2012), and therefore multifractal analysis applied to spike train patterns may quantify a possible basis of memory detected as multifractal complexity.

To assess if active memory processing is reflected by multifractal measures, analyses of monofractal long-range temporal correlations (LRTCs) and multifractal complexity in hippocampal interspike interval (ISI) neural sequences were conducted during a working memory task. *In vivo* electrophysiological recordings of rat hippocampal CA3 and CA1 subregions were conducted during a resting state condition in a neutral (task-independent) context for 25–30 min both before and after performance of the delayed nonmatch-to-sample (DNMS) task. Between the pre-task recording and the DNMS task, rats were injected with vehicle or tetrahydrocannabinol (THC), a psychoactive component of cannabis known to impair memory function (Hampson and Deadwyler, 2000). Prior results demonstrated that hippocampal neurons with memory-correlated firing rate alterations (functional cell types, FCTs) recorded during the DNMS task were more multifractal than non-memory neurons (non-FCTs) and THC administration impaired memory while reducing multifractality (Fetterhoff et al., 2015). By examining the same neurons before, during and after the DNMS task, alterations in multifractality were assessed in a different context. These experiments and analyses were designed to extend previous findings by testing three new hypotheses and facilitating a stronger intuition concerning multifractal properties of hippocampal microcircuits. First, we hypothesized that LRTCs, as indicated by the Hurst exponent, would decrease during the DNMS task compared to resting (pre/post) recording conditions. Since LRTCs arise when distant activity has a greater influence on future activity patterns, we hypothesized a decrease would occur due to the constantly changing requirements of the DNMS task. Second, we hypothesized that an increase in multifractal complexity reflects active memory processing in populations of hippocampal neurons and therefore, spike trains should be more multifractal during the DNMS task compared to both pre- and post-task recording phases. Third, we hypothesized that THC would decrease both multifractal complexity and Hurst exponents during the task and post-recording phases compared to vehicle control recordings during the same phase. Finally, to enrich conceptual interpretation of multifractal complexity and LRTCs and establish the difference between structure (multifractality) and amount of variability, classical spike train variability measures (coefficient of variation, ISI STD and mean ISI) were compared with the mono- and multifractal variables.

The primary goal of this study was to assess the capacity of multifractal analysis to distinguish between recording phases and drug conditions. Fourier analysis of neuronal signals is one commonly employed method to distinguish between physiological and cognitive states (De Carli et al., 2004; Jones and Wilson, 2005; Nguyen et al., 2008; Palva et al., 2010; Van Someren et al., 2011; Garn et al., 2014), and therefore, the performance of multifractal analysis was compared to the frequency content computed from the same spike trains. The results showed that the monofractal Hurst exponent and magnitude of multifractality could differentiate between more recording/drug conditions compared to frequency content (theta and delta) and further support utility of multifractal analysis for this objective (Weiss et al., 2009; Zorick and Mandelkern, 2013). Multifractal analysis has the potential to generate novel insights into the role of neuronal ensembles by quantifying different temporal features compared to other analyses.

## Materials and methods

### Rats

Male Long-Evans rats (Harlan) aged 6–10 months (*n* = 10) were tested under protocols approved by the Wake Forest University Institutional Animal Care and Use Committee, and in accordance with the Association for Assessment and Accreditation of Laboratory Animal Care and the National Institute of Health Guide for the Care and Use of Laboratory Animals (NIH Publication No. 8023). All animals were individually housed and allowed free access to food with water regulation to maintain 85% of *ad libitum* body weight during testing. Upon termination of the study, all rats were anesthesized with ketamine (100 mg/kg) and brains were perfused with formaldehyde for preservation and subsequent histology to confirm electrode placement.

#### Apparatus

The behavioral testing apparatus for the delayed nonmatch to sample (DNMS) task as used in other studies (Hampson and Deadwyler, 2000; Hampson et al., 2012) consisted of a 43 × 43 × 50 cm Plexiglas chamber with two retractable levers (left and right) positioned on either side of a water trough on the front panel. A nosepoke device (photocell) was mounted in the center of the wall opposite the levers with a cue light positioned immediately above the nosepoke device. A video camera was mounted on the ceiling and the entire chamber was housed inside a commercially built sound-attenuated cubicle.

#### Delayed nonmatch-to-sample (DNMS) task

**Figure 1:**
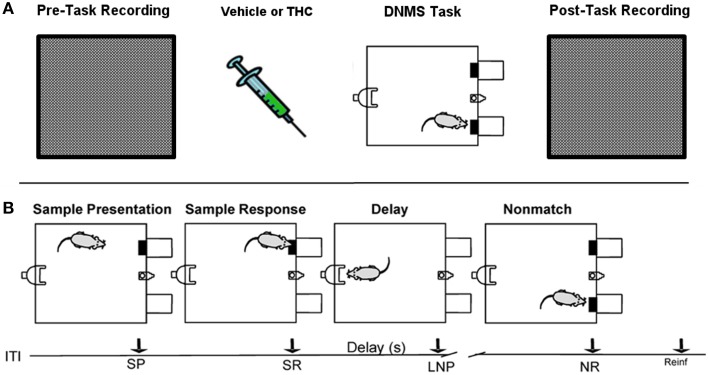
**Rest (pre/post) and Delayed Nonmatch-to-Sample (DNMS) task recording paradigm**. **(A)** Prior to each testing session, all rats were recorded in a white, rectangular plastic box for 25–30 min (pre-task recording). Upon completion of pre-task recording phase, the same rats were injected with either vehicle or delta-9-tetrahydrocannabinol (THC) 5–10 min before the start of delayed nonmatch-to-sample (DNMS) task. Immediately after completing the DNMS task, rats were put back into the same plastic chamber for another 25–30 min recording (post-task recording). **(B)** Progression of the DNMS task is illustrated. A 10 s Intertrial Interval (ITI) precedes the Sample Presentation (SP). Rats must make the Sample Response (SR) and remember the lever position throughout the variable 1–30 s delay that terminates after the Last Nosepoke (LNP). The LNP signals extension of both levers and rats receive a water reward (reinforcement) for appropriately making a Nonmatch Response (NR).

The DNMS task consisted of three main phases: Sample, Delay and Nonmatch (Figure 1B). The Sample phase initiated the trial via presentation of either the left or right lever (50% probability), which required the animal to press and make the Sample Response (SR). The lever was then retracted and the Delay phase of the task initiated, as signaled by the illumination of a cue light over a nosepoke photocell device on the wall opposite to where the lever was presented. At least one nosepoke (NP) was required following the interposed delay interval which varied randomly in duration (1–30 s) on each trial during the session. The Nonmatch phase began when the delay timed out, the photocell cue light turned off, and both the left and right levers on the front panel were extended. Correct responses consisted of pressing the lever in the Nonmatch phase located in the spatial position opposite to the position of the SR; in other words, a Nonmatch response (NR). This produced delivery of a 0.4 ml water reward in a reservoir between the two levers. After the NR the levers were retracted for a 10.0 s intertrial interval (ITI) before the Sample lever for the next trial was presented. A lever press at the same position as the SR (match response) constituted an “error” with no water delivery and turned off chamber house lights for 5.0 s with the next trial presented 5.0 s later. Individual performance was assessed as % NRs (correct responses) with respect to the total number of trials (80–100) per daily (1 h) session.

#### Task and rest (non-task) recording paradigm

All rats were recorded for 25–30 min in a bare, white 38 × 29 × 30 cm plastic container that was inserted within the DNMS testing chamber both before (pre) and after (post) the DNMS task (Figure 1A). This constituted a neutral environment used to record task-independent neuronal activity that was opaque to prevent rats from seeing task components, such as levers or the nosepoke device.

### Drug preparation and administration

Δ^9^-THC was obtained from the National Institute on Drug Abuse as a 50 mg/ml solution in ethanol. Detergent vehicle was prepared from Pluronic F68 (Sigma, St. Louis, MO), 20 mg/ml in ethanol. Δ^9^-THC was added to the detergent-ethanol solution (0.04–0.12 ml of THC), and then 2.0 ml of saline (0.9%) was slowly added to the ethanol-drug solution. The solution was stirred rapidly and placed under a steady stream of nitrogen gas to evaporate the ethanol (~10 min). This resulted in a detergent-drug suspension (12.5 mg/ml THC), which was sonicated and then diluted with saline to final injection concentrations (1.0–3.0 mg/ml THC). On testing days, animals were injected intraperitoneally with the detergent vehicle solution or the THC drug-detergent solution (1 ml/kg) immediately after the pre-task recording phase and approximately 5–10 min before the start of the behavioral session. At least one vehicle day was imposed between each drug-testing day. All rats received THC on 5–8 days. All drug solutions were mixed fresh each day.

#### Hippocampal electrode array surgery

All surgical procedures conformed to National Institutes of Health and Association for Assessment and Accreditation of Laboratory Animal Care guidelines, and were performed in a rodent surgical facility approved by the Wake Forest University Institutional Animal Care and Use Committee. Electrode arrays and recordings were the same as described in several prior publications from this laboratory (Hampson et al., 1999, 2012; Hampson and Deadwyler, 2000). After being trained to criterion performance level in the DNMS task animals were anesthetized with ketamine (100 mg/kg) and xylazine (10 mg/kg) and placed in a stereotaxic frame. Craniotomies (5 mm-diameter) were performed bilaterally over the dorsal hippocampus to provide for implantation of 2 identical array electrodes (Neurolinc, New York, NY), each consisting of two rows of 8 stainless steel wires (diameter: 20 μm) positioned such that the geometric center of each electrode array was centered at co-ordinates 3.4 mm posterior to Bregma and 3.0 mm lateral (right or left) to midline (Paxinos and Watson, 1997). The array was designed such that the distance between two adjacent electrodes within a row was 200 μm and between rows was 400 μm to conform to the locations of the respective CA3 and CA1 cell layers. The longitudinal axis of the array of electrodes was angled 30° to the midline during implantation to conform to the orientation of the longitudinal axis of the hippocampus, with posterior electrode sites more lateral than anterior sites. The electrode array was lowered in 25–100 μm steps to a depth of 3.0–4.0 mm from the cortical surface for the longer electrodes positioned in the CA3 cell layer, leaving the shorter CA1 electrodes 1.2 mm higher with tips in the CA1 layer. After placement of the array the cranium was sealed with bone wax and dental cement and the animals treated with Ketoprofen (3.0–5.0 mg/kg) for pain relief over the next 4–6 h. The scalp wound was treated periodically with Neosporin antibiotic and systemic injection of penicillin G (300,000 U, intramuscular) were given to prevent infection. Animals were allowed to recover from surgery for at least 1 week before continuing behavioral testing (Berger et al., 2011; Hampson et al., 2012).

#### Electrophysiological monitoring, acquisition and waveform sorting of neuronal data

Animals were connected by cable to the recording apparatus via a 32-channel headstage and harness attached to a 40-channel slip-ring commutator (Crist Instruments, Hagerstown, MD) to allow free movement in the behavioral testing chamber. Single neuron action potentials (spikes) were isolated by time-amplitude window discrimination and computer-identified individual waveform characteristics using a multi-neuron acquisition (MAP) processor (Plexon Inc., Dallas, TX, USA). Single neuron spikes were recorded daily and identified using waveform and firing characteristics within the task (perievent histograms) for each of the DNMS events (SR, LNP, and NR). To maintain waveform shape across days, all recorded data was concatenated into one file (separately for each rat) and offline sorting was performed using principal component analysis, peak-valley, and nonlinear energy algorithms in Offline Sorter (Figure S1; Plexon Inc., Dallas, TX, USA). Hippocampal neuron ensembles used to distinguish recording phases and drug treatment conditions consisted of 10–30 single neurons, each recorded from a separate identified electrode location on either of the bilateral arrays. Only isolated neural spike waveforms exhibiting firing rates of CA1 and CA3 principal cells (i.e., 0.5–8.0 Hz average firing rate) and consistent multifractal properties across sessions were included in analyses. Previous work has shown that hippocampal neurons recorded identified in this manner exhibit consistent mean, baseline, and DNMS task modulated firing rate over multiple task sessions (Deadwyler et al., 1996; Hampson et al., 1999, 2003).

### Multifractal detrended fluctuation analysis (MFDFA)

**Figure 2:**
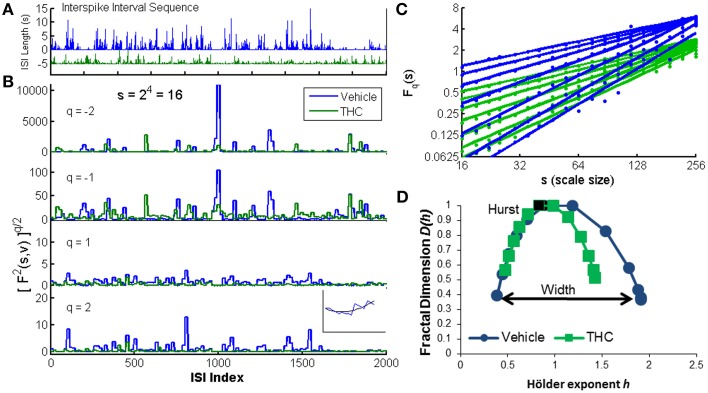
**Multifractal detrended fluctuation analysis**. An illustration of the MFDFA method was created using one CA1 neuron recorded on two different days: one vehicle condition (blue) and one tetrahydrocannbinol (THC) condition (green). **(A)** The interspike interval sequence (ISI) of each neuron is shown for the first 2000 ISIs. Five seconds were subtracted from the entire THC ISI sequence for illustration purposes only. **(B)** The fluctuation function *F* is shown at scale 16 (*s* = 16) for four different q-order statistical moments. Negative q-order statistical moments amplify small fluctuations, while positive moments amplify large fluctuations. Inset: *F*^2^(*v, s*) is the root mean-squared residual between the fit *y*_*v*_ (black) of one segment *s* from the walk-like time series *Y* (blue). **(C)** The changes in variability across scales are indicated by variable slopes at different qth powers (integer q-values from −3 to 3). The q-order Hurst exponent *H(q)* is the slope of each regression line. Blue lines are from the vehicle recording and green lines are from the THC recording. Dots indicate individual values from each scale (19 scales ranging from 16 to 256). **(D)** Multifractal complexity is visualized with the multifractal singularity spectrum. The Hurst exponent is closely related to the *h*-value at the apex of the singularity spectrum (black data points). The width is obtained by subtracting *h*-values at each end of the spectrum (independent of *D(h)* values) indicated by the black arrow. The singularity spectrum for the vehicle condition is wider than the THC condition, indicating THC decreases multifractality.

Detailed descriptions of multifractal detrended fluctuation analysis (Kantelhardt et al., 2002; Kantelhardt, 2012) and associated Matlab code (Ihlen, 2012) are available elsewhere. We briefly summarize and illustrate the main components of the MFDFA method. A demonstration of this method (Figure 2) was constructed using recordings of a single neuron (Figure S1) recorded during the DNMS task over two sessions/days: one vehicle (blue) and one THC (green). First, a neuronal spike train is converted to a sequence of interspike intervals (ISIs; Figure 2A), represented as *x*, and commonly referred to as a noise-like time series (Ihlen, 2012). The ISIs are converted into a “random walk-like” time series *Y(i)* by subtracting the mean and integrating the ISI signal *x*:

(1) $$Y(i)=\sum_{k\;=\;1}^{i}\left[x_{k}-\langle x\rangle \;\right],i=1,\ldots ,N$$

The random walk *Y(i)* (Figure 2B inset, blue line) is divided into *N*_*s*_ non-overlapping segments of equal length *s* (scale). The local quadratic trend *y*_*v*_ (Figure 2B inset, black line) is calculated for each of the *N*_*s*_ segments *v* by a least-square fit of the series to determine the local root mean square variation *F* for each segment *v* (Figure 2B):

(2) $$F^{2}(v,s)=\frac{1}{s}\sum_{i\;=\;1}^{s}{\left\{Y\left[\left(v-1\right)s+i\right]-y_{v}\left(i\right)\right\}}^{2}$$

In this way, *F*^2^(*v, s*) is essentially the mean-square error difference between the quadratic fit *y*_*v*_ and the walk-like ISI sequence *Y*. Visualization of the walk like ISI sequence at multiple q-order statistical moments permits observation of the structure of variability quantified by multifractal analysis (Figure 2B). Periods of low activity (i.e., clusters of short ISIs, faster firing rate) are amplified with negative moments (Figure 2B). Conversely, periods of high activity (i.e., clusters of longer ISIs, slower firing rate) are amplified with positive moments (Figure 2B, bottom). Next, the qth order fluctuation function is determined by averaging over all segments *v* for each qth power (Figure 2C):

(3) $$F_{q}\left(s\right)={\left\{\frac{1}{Ns}\sum_{v\;=\;1}^{Ns}{\left[F^{2}\left(v,s\right)\right]}^{q/2}\right\}}^{1/q}$$

The scaling behavior of the fluctuation function is seen by analyzing log-log plots of *F*_*q*_(*s*) vs. s for each qth power (Figure 2C). Standard (monofractal) Detrended Fluctuation Analysis calculates the Hurst exponent from the slope of the power-law regression line between the overall root mean square variation *F* across multiple scales, *s*, for a single statistical moment, *q* = 2. Multifractal analysis performs the same linear regression for a broad range of statistical moments *q*. *F*_*q*_(*s*) increases as a power-law for large values of *s* if the signal *x* contains long-range temporal correlations (LRTCs):

(4) $$F_{q}\left(s\right)\;\sim \;s^{H(q)}$$

The Hurst exponent (Hurst, 1951) is the slope of *F*_*q*_(*s*) on this log-log plot for *H*(*q* = 2). A greater slope yields a larger Hurst exponent and indicates increased LRTCs which defines how fast the overall root mean square variation grows with increasing segment size (scale = s). Hurst exponents greater than 0.5 indicate the time series contains positive correlations (i.e., persistent structure), Hurst exponents ranging from 0 to 0.5 indicate negative correlations and uncorrelated Gaussian noise has a Hurst exponent equal to 0.5. In multifractal signals, changes in LRTCs occur at different q-order statistical moments. These variations are visualized by comparing the q-order Hurst exponents (slopes of regression lines) on log-log plots of *F*_*q*_(*s*) vs. scale *s*, where each line is computed from a different (integer) qth power ranging from −3 to 3 (Figure 2C). Our example shows more variable slopes in the vehicle condition (Figure 2C, blue) compared to the similar slopes in THC condition (Figure 2C, green), indicating greater multifractality during the vehicle condition. The qth order fluctuation function *F*_*q*_(*s*) is one way to visualize multifractal properties of variability, but generally the multifractal singularity spectrum (Figure 2D) is constructed to illustrate the distinction between (mono)-fractal and multifractal analyses. The q-order Hurst exponent *H(q)* is converted to the q-order mass exponent τ(q):

(5) τ(q)=qH(q)−1

Finally, a Legendre transform relates τ(q) to the fractal dimension *D(h)* and Hölder exponent *h*:

(6) $$h=t^{\prime }(q)\text{ and }D(h)=qh-\tau \left(q\right)$$

The singularity spectrum is like a histogram of the q-order Hurst exponents (slopes of regression lines in Figure 2C). The magnitude of multifractality is determined by the width of the singularity spectrum and consequently the range of Hölder exponents *h* covered by the ISI signal (Figure 2D). The Hurst exponent is closely approximated by the Hölder exponent at the apex of the singularity spectrum (where *D*(*h*) = 1). In our example, the singularity spectrum for the THC condition is narrower, and thus less multifractal than the singularity spectrum computed for the control condition (Figure 2D). The parameters used for all analyses were determined by viewing log-log plots of *F*_*q*_(*s*) vs. s for each qth power and multifractal singularity spectra that resembled those found in the literature (Kantelhardt et al., 2002; Ihlen, 2012). MFDFA was performed by fitting a second-order polynomial, scales used ranged from 16 to 256, and singularity spectrum width was determined by computing *h*(*q* = 3)−*h*(*q* = −3). All analyses were performed in Matlab using publicly available MFDFA code (Ihlen, 2012).

### Fourier transform

A fast Fourier transform of the spike train data in 1 ms binary bins where 1 = spike and 0 = no spike was performed for every neuron recorded during every recording phase. Delta and theta power were measured by taking the ratio of signal power in the delta (0.5–4.0 Hz) or theta range (4–8 Hz) over the total power of the normalized signal, bandpass filtered from 0.5 to 12 Hz. All analyses were performed using Matlab.

### Statistical analyses

All calculations were performed in Matlab, results were recorded spreadsheets and imported into Statistical Analysis Systems (SAS) software (SAS Institue, Cary, NC) to perform repeated measures ANOVAs and correlations. A total of seven repeated measures ANOVAs were performed using these dependent variables: the Hurst exponent, singularity spectrum width, coefficient of variation, ISI standard deviation, mean ISI, delta power, and theta power. Neurons were the subject identifier, each session as a within-subjects effect, and no group identifier was used. The covariance structure used was compound symmetry. Main effects of drug condition or recording phase were only discussed when the interaction between drug condition and recording phase was non-significant. When significant ANOVA effects were found, *post-hoc* tests were performed with 10,000 Monte Carlo permutations to determine two-tailed *p*-values. Significance levels were set to *p* < 0.0083 to control for multiple comparisons (Bonferoni correction). Correlations (Spearman's rho) were used to examine the associations between mono- and multifractal variables (Hurst exponent and singularity spectrum width) and coefficient of variation, ISI standard deviation, mean ISI, delta and theta power. 55 total correlations were performed to assess the relationships for monofractal and multifractal variables during all three recording phases (pre-task, task, post-task).

## Results

To investigate dynamical interspike interval (ISI) patterns associated with different hippocampal microcircuit processing states, a comparision was made between those generated during the DNMS task (Deadwyler et al., 1996; Hampson et al., 1999) and those occuring during a resting state after either vehicle or THC administration (Hampson and Deadwyler, 2000). Hippocampal principal (pyramidal) cells were identified based on mean firing rate (0.5–8.0 Hz), and neurons exhibiting rates outside this range were excluded. Identified neurons from selected wires were “tracked” from day to day by waveform and multifractal properties (i.e., Hurst exponent and width). A total of 197 hippocampal principal (pyramidal) cells were recorded from 10 different rats and each neuron was recorded from the same electrode over multiple days (10–16 recording days per rat). 117 CA1 and 80 CA3 neurons were analyzed for this study. Every neuron included in the analyses was recorded during at least 4 days (2 vehicle and 2 THC).

### Delayed nonmatch-to-sample task

**Figure 3:**
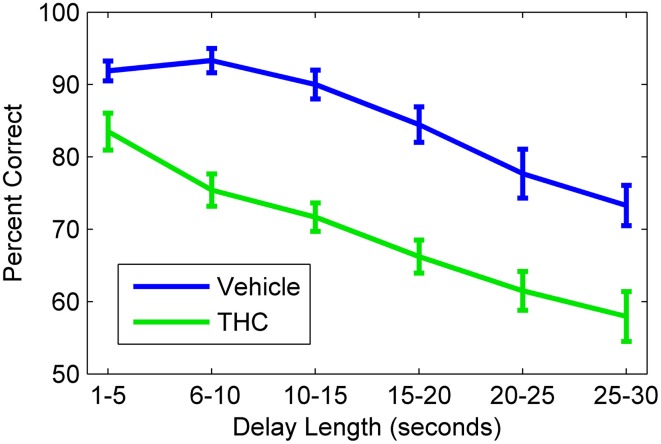
**Delayed nonmatch-to-sample behavioral performance during vehicle and tetrahydrocannabinol (THC) sessions**. Mean correct nonmatch responses summed across all rats (*n* = 10) shows the delay-dependent decline in performance under both conditions. A within subjects design with at least one non-drug day between THC administration was used. All animals were given THC (1.0–3.0 mg/kg) for at least five sessions spaced over consecutive weeks. Error bars indicate S.E.M.

Hippocampal spike trains were recorded during a resting state condition in a neutral (i.e., task-independent) environment both before (pre) and after (post) the DNMS task (Figure 1A) to assess the influence that active memory processing (during the task) exerts on the structure of spike train variability, as indicated by multifractal analysis. This approach was designed to assess electrophysiologcal distinctions between three different recording phases (pre-task, task, post-task) and in two drug conditions. Since drug injections (pluronic vehicle or THC) were given immediately after the pre-task/pre-drug resting state recording phase, all computed measures for the pre-task phase are equal across the two drug conditions. THC, the main active ingredient in cannabis (Gaoni and Mechoulam, 1964), was chosen because it impairs memory encoding during the DNMS task (Hampson and Deadwyler, 1999, 2000), reduces LRTCs and multifractal complexity of task-related neuronal spike trains (Fetterhoff et al., 2015) and impairs theta frequency-related working memory performance in both rats (Robbe et al., 2006) and humans (Ilan et al., 2004; Böcker et al., 2010). THC doses were chosen to maximally impair DNMS performance in order to examine effects on associated multifractal spike train characteristics using previously established dose-response relationships (Hampson and Deadwyler, 2000). Working memory was assessed in 10 rats after vehicle or THC administration using the DNMS task (Figure 1B). A within subjects design was used to assess behavioral performance and hippocampal electrophysiology for 5–8 days per drug condition (vehicle or THC) per rat. The DNMS performance was inversely correlated with delay length, as all animals performed worse at longer delays (Figure 3). THC (green line) impaired performance compared to vehicle (Figure 3, blue line).

### Frequency and singularity spectra of hippocampal spike trains

Interactions between frequency bands are believed to represent coordination and exact timing relationships within neuronal ensembles (Buzsáki and Moser, 2013). Multifractal dynamics are believed to arise from interactions across spatiotemporal scales (Ihlen and Vereijken, 2010; Kelty-Stephen et al., 2013) and therefore may represent phenomena related to frequency-specific activity. Additionally, both analyses are commonly used to assess differences in sleep/arousal state, and increasing interest is focused on multifractal analysis as an automatic sleep stage detection method (Weiss et al., 2009; Zorick and Mandelkern, 2013). The performance of multifractal and Fourier analyses was evaluated here in terms of their ability to distinguish between recording phases and drug treatment conditions (Figure 1) based on analyses of hippocampal spike trains. Fourier transforms were used to measure both delta (0.5–4 Hz) and theta (4–8 Hz) power in binary spike train representations. These frequency bands were chosen due to their prominence during the DNMS task (Hyman et al., 2010, 2011) and pervasive presence in working memory literature (Sato and Yamaguchi, 2003; Mormann et al., 2008; Assenza et al., 2013; Clemens et al., 2013; Hasselmo and Stern, 2014). MFDFA quantifies the structure of variability of an ISI sequence and permits estimation of the multifractal singularity spectrum (Ihlen, 2012) that describes a given spike train. The singularity spectrum presents two important pieces of information: the Hurst exponent and magnitude of multifractality. The Hurst exponent *H* is a self-similarity parameter that quantifies long-range temporal correlations (LRTCs) and takes values nearly equivalent to the Hölder exponent *h*-value at the center apex of the singularity spectrum (Figure 2D) where *D(h)* = 1 (Ciuciu et al., 2012; Ihlen, 2012). The Hurst exponent quantifies the (mono)-fractal structure of the ISI time series, however, many physiological signals exhibt a wider range of dynamical activity that is better described as multifractal. The magnitude of multifractality quantifies the structure of variability in an interspike interval (ISI) sequence and is directly proportional to the width of the singularity spectrum. The width is defined by the range of local Hölder exponents *h* (Figure 2D) which quantify the dynamical profile of a time signal.

**Figure 4:**
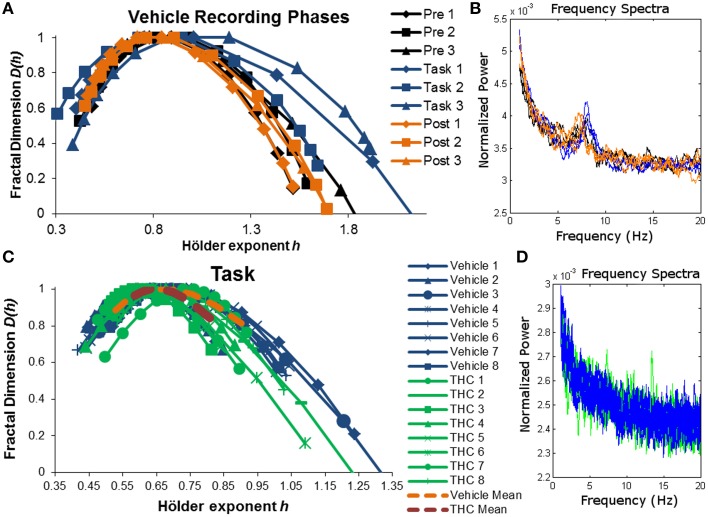
**Singularity and frequency spectra of example neurons**. Singularity and frequency spectra pairs are shown for two different CA1 neurons. Single neurons were measured across multiple days in various recording phases (pre-task, task, post-task) and drug conditions (vehicle or THC) and each spectrum trace represents the multifractal complexity **(A,C)** and frequency content **(B,D)** computed from one recording phase on 1 day. **(A)** Singularity spectra are shown for each recording phase over three vehicle experiment days (diamonds for day 1, squares for day 2, triangles for day 3). Singularity spectra are wider, thus multifractal complexity is greater, during the task compared to either resting state recording phases. **(B)** Frequency spectra are shown for the same neuron recorded during the same 3 days as in **(A)** and color coded to match the legend in **(A)**. This neuron exhibits both delta (0.5–4 Hz) and theta (4–8 Hz) frequency activity during all recording phases on all days. **(C)** Singularity spectra computed from the DNMS task are compared between vehicle and THC conditions for one example neuron recorded over 16 total days. Individual session singularity spectra are plotted in thin blue lines for vehicle and green lines for THC sessions. The average singularity spectra for this neuron is plotted as a dashed orange line for vehicle and as a dashed red line for THC. **(D)** Frequency spectra are shown for the same neuron recorded during the same 16 days as in **(C)** and color coded to match the legend in **(C)**. Only spectra from individual neurons were plotted. This neuron exhibits delta rhythm only.

**Figure 5:**
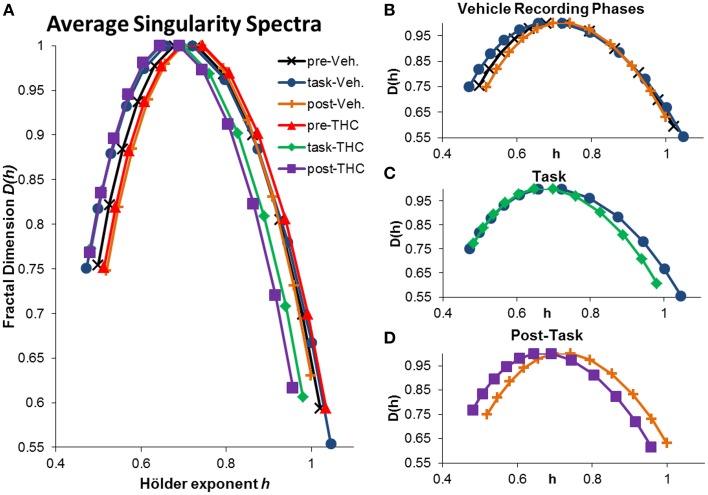
**Average singularity spectra across recording phases and drug conditions**. Average spectra were computed by averaging all neurons within the respective recording phase and drug condition. A total of 197 hippocampal neurons were recorded from 10 different rats. Each neuron was recorded from the same electrode over multiple days and multifractal analysis was performed on a total of 5143 individual ISI sequences. 771–1004 individual ISI sequences were averaged for each condition. The legend in the upper right corner of **(A)** holds true for all figures. **(A)** Average singularity spectra were obtained from all neurons recorded during their respective recording phase and drug condition. **(B)** Average singularity spectra from all recording phases during vehicle treatment are plotted for comparison. Neurons exhibit greater multifractal complexity (i.e., wider singularity spectra; wider range of Hölder exponents *h*) during the task compared to either resting state. Long-range temporal correlations, indicated by the Hurst exponent, which is closely related to the Hölder exponent at the apex of the singularity spectrum [where *D*(*h*) = 1], are stronger during the resting states compared to the task. **(C)** Average singularity spectra from both drug conditions during DNMS task recordings show that THC reduces multifractal complexity, as indicated by decreased singularity spectra width. **(D)** Average singularity spectra taken from post-task recording phases show that THC reduces LRTCs (i.e., decreased Hurst exponent) compared to vehicle recordings; this effect is seen as the leftward shift in the THC spectrum comapred to the vehicle one. Multifractal complexity was unchanged by THC during post-task recordings.

**Figure 6:**
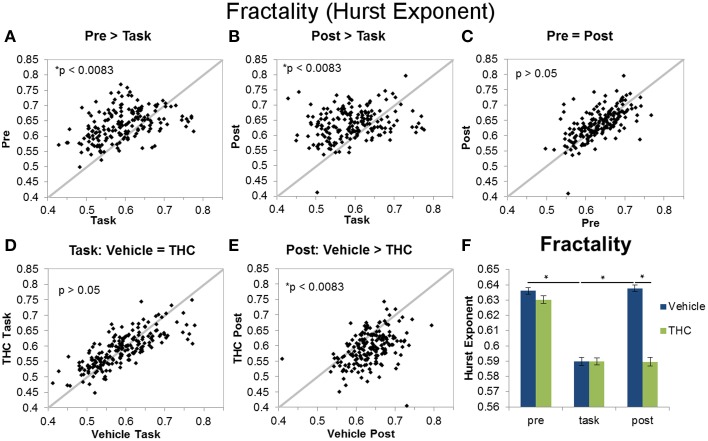
**Fractality (Hurst exponent) across recording phases and drug conditions**. The scatter plots show each data point obtained by averaging the Hurst exponent for individual neurons over all recordings. All neurons included in the analysis were recorded for 2–8 days of vehicle and THC administration. Thick gray lines are y = x. Data points from vehicle only sessions are displayed in **(A–C)**. **(A)** Neurons exhibited greater Hurst exponents during the pre-task recordings compared to DNMS task recordings. **(B)** The Hurst exponent of ISIs was also greater during the post-task compared to task recordings. **(C)** Hurst exponents were similar during pre-task and post-task recordings. **(D)** THC had no effect on the Hurst exponent of neurons recorded during the DNMS task. **(E)** THC significantly reduced the Hurst exponent during post-task recordings. **(F)** Each bar was obtained by averaging Hurst exponent values from individual spike trains within specified recording phase and drug treatment combinations (*n* = 771–1004 neurons per group). Errors bars represent S.E.M. Statistical significance is designated by ^*^ indicating *p* < 0.0083.

**Figure 7:**
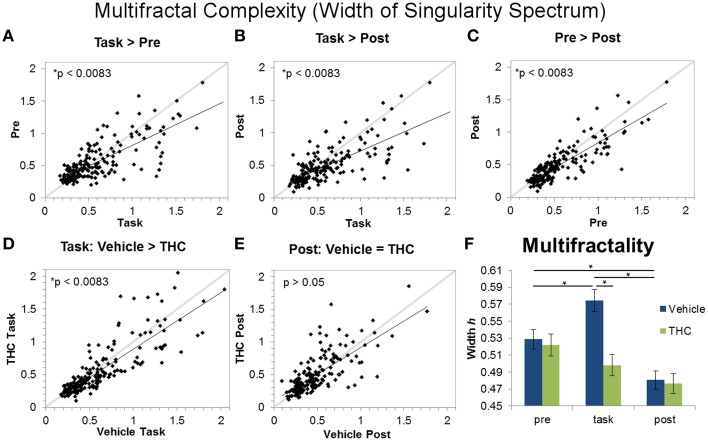
**Multifractality (singularity spectrum width) across task and rest conditions after vehicle or THC administration**. The scatter plots show each data point obtained by averaging singularity spectrum width for individual neurons over all recordings. All neurons included in the analysis were recorded for 2–8 days of vehicle and THC administration. Thick gray lines are y = x and thin black lines are regression lines. Data points from vehicle only sessions are displayed in **(A–C)**. **(A)** Neurons exhibit greater multifractal complexity (defined by the width or the singularity spectrum) during the DNMS task compared to pre-task recordings. **(B)** Multifractal complexity of ISIs is greater during the task compared to post-task recordings. **(C)** On average, multifractal complexity is greater during the pre-task resting state recording compared to the post-task recordings. **(D)** THC significantly reduces multifractal complexity during the DNMS task. **(E)** THC has no effect on multifractal complexity during the post-task recordings. **(F)** Each bar was obtained by averaging multifractality (width *h*) from individual spike trains within specified recording phase and drug treatment combinations (*n* = 771–1004 neurons per group). Errors bars represent S.E.M. Statistical significance is designated by ^*^ indicating *p* < 0.0083.

**Figure 8:**
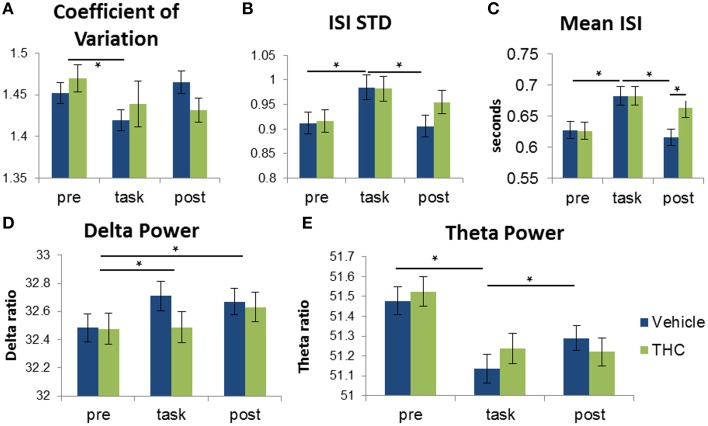
**Distinction between recording phases and drug conditions using frequency spectra and spike train variability measures**. Each bar was obtained by averaging values from individual spike trains within specified recording phase and drug treatment combinations (*n* = 771–1004 neurons per group). Vehicle (blue) or THC (green) was given after pre-task recording, so measures obtained during the pre-task recording should be the same for drug conditions. Statistical significance is designated by ^*^ indicating *p* < 0.0083 (Bonferoni correction). Error bars represent S.E.M. **(A)** Repeated measures ANOVA using coefficient of variation (CV) as the dependent variable yielded a significant main effect of recording phase but no significant interaction between drug condition and recording phase. CV was greater during pre-task recordings compared to recordings during the DNMS task. **(B)** A significant interaction between recording phase and drug condition revealed that ISI standard deviation is greater during the task compared to pre- and post-task recordings. **(C)** A significant interaction between recording phase and drug condition revealed that ISIs recorded under vehicle administration were larger during the task vs. pre- and post-task conditions. THC increased mean ISI only during the post-task recording but had no effect during the task. **(D)** A significant main effect of recording phase was found when assessing delta power: Delta power was larger during task and post-task vs. pre-task sessions. **(E)** Theta power of hippocampal neurons was higher during the task-independent (pre- and post-task) resting phases compared to during DNMS task performance.

The singularity and frequency spectra for two different example neurons are shown in Figure 4 while the population singularity spectra are shown in Figure 5. Repeated measures ANOVA results from population analyses are briefly mentioned here and presented fully in the subsequent sections. The first example permits comparision of all three recording phases taken from vehicle treatment conditions (Figures 4A,B, 5B). One example neuron exhibits increased multifractal complexity during the DNMS compared to either resting state recordings (Figure 4A). The frequency spectra for this same neuron exhibits both delta and theta power in all recording phases (Figure 4B). This neuron illustrates the same effect found in the population (Figure 5B): multifractal complexity (width) increases from post-task to pre-task to task (Figure 7F) and LRTCs (Hurst exponent) are larger during the resting states (pre- and post-task) compared to the task (Figure 6F). Although the singularity spectra are discernable across task phases for this neuron, the frequency spectra were not (Figure 4B). However, the population analyses revealed increased theta power during vehicle resting state recordings (pre- and post-task) compared to vehicle task recordings (Figure 8E).

Another example neuron was chosen to illustrate the finding that THC reduced multifractal complexity (width) during the DNMS task compared to vehicle (Figure 4C). This neuron was recorded over 16 total days, 8 vehicle days and 8 THC days. Individual and average singularity spectra are shown (Figure 4C). The frequency spectrum for this neuron shows delta rhythm but not theta (Figure 4D). This neuron is representative of the entire population: during the DNMS task, THC reduces multifractality (width; Figures 5C, 7D,F), but has no significant effect on the Hurst exponent (Figures 6D,F). Additionally, THC did not affect frequency content during the DNMS task compared to vehicle (Figures 8D,E).

### Temporal correlations and multifractal complexity of hippocampal population

Two repeated measures ANOVAs were performed to assess the capacity of monofractal and multifractal variables, the Hurst exponent and singularity spectrum width, to distinguish between recording phases and drug treatment conditions. Hurst and width were used as dependent variables to examine main effects of drug condition (vehicle vs. THC) and recording phase (pre-task/pre-drug, DNMS task, post-task) and an interaction between the two. Only interactions (not main effects) are discussed when significant. We primarily wanted to evaluate the ability of computed variables to establish 6 *ad hoc* chosen differences: vehicle pre-task vs. task, vehicle task vs. post-task, vehicle pre-task vs. post-task, vehicle task vs. THC task, vehicle post-task vs. THC post-task, and THC task vs. THC post-task. Since the distributions were long-tailed, *post-hoc* Monte Carlo permutation tests were performed with signifiance levels set at *p* < 0.0083 (Bonferoni correction). All analyses were performed on a total of 5143 individual interspike interval (ISI) sequences: 829 (495 CA1, 334 CA3) individual ISI sequences from vehicle pre-task recordings, 1004 (592 CA1, 412 CA3) from vehicle task recordings, 848 (510 CA1, 338 CA3) from vehicle post-task recordings, 771 (443 CA1, 328 CA3) from THC pre-task/pre-drug recordings, 923 (550 CA1, 373 CA3) from THC task recordings, and 768 (453 CA1, 315 CA3) from THC post-task recordings. Population statistics were performed for all hippocampal neurons (Figures 6, 7) and differences by hippocampal location were shown in the Supplementary Material (Figure S2).

The Hurst exponent is a monofractal self-similarity parameter that quantifies LRTCs in an ISI sequence. A significant main effect of drug condition [*F*_(1, 196)_ = 97.61, *p* < 0.0001], a significant main effect of recording phase [*F*_(2, 379)_ = 182.7, *p* < 0.0001], and a significant interaction [*F*_(2, 369)_ = 80.77, *p* < 0.0001] were found when using the Hurst exponent as the dependent variable. The significant drug treatment by recording phase interaction revealed three out of six important group differences (Figure 6F): both pre- and post-task vehicle recordings contain greater LRTCs than task recordings (Figures 6A,B) and THC reduces LRTCs compared to control during the post-task recording (Figure 6E). No significant difference was found between the Hurst exponent from vehicle pre-task recordings compared to vehicle post-task recordings (Figure 6C), nor was a difference established between the Hurst exponent computed from vehicle or THC task recordings (Figure 6D). After THC treatment, the Hurst exponent of the task condition was similar to those from the post-task (Figure 6F).

Multifractal complexity reflects energy flow through all scales of a dynamical system (Dixon et al., 2012; Kelty-Stephen et al., 2013) and is greater in memory processing neurons compared to randomly spiking ones (Fetterhoff et al., 2015). Therefore, multifractality may selectively arise in hippocampal microcircuit processing when task-specific input-output transformations occur (Berger et al., 2012; Hampson et al., 2012). A repeated measures ANOVA using multifractal width as the dependent variable yielded a significant main effect of drug condition [*F*_(1, 196)_ = 11.09, *p* = 0.001], a significant main effect of recording phase [*F*_(2, 379)_ = 30.8, *p* < 0.0001], and a significant interaction [*F*_(2, 369)_ = 13.87, *p* < 0.0001]. The significant drug condition by recording phase interaction established four out of six significant differences (Figure 7F): in the vehicle condition, all three recording phases are different from each other (Figures 7A–C; task > pre > post). Multifractal complexity was greatest when hippocampal ensembles were processing task-relevant information. THC reduced multifractal complexity (width) during the task (Figure 7D) but had no effect on multifractal complexity during post-task recordings (Figure 7E). Therefore, the effect of THC to reduce multifractality of hippocampal neurons only occurred when memory was impaired during the task. Additionally, multifractal complexity (width) of neurons after THC administration was similar during DNMS task and post-task phases (Figure 7F). The lack of multifractality adjustments between task and post-task recordings after THC administration suggests that THC impairs functional transistions in hippocampal microcircuit activity detected as multifractal complexity.

### Standard variability measures are marginally task-specific

Multifractal analysis quantifies the structure of variability (Ihlen, 2012); therefore, to establish the differences between structure and amount of variability, we compared multifractal indices to standard variability measures of the same hippocampal multi-neuron data. To determine if the amount of variability of hippocampal spike trains can account for distinctions between recording phases and drug conditions, the coefficient of variation (CV) was computed for all hippocampal ISI sequences by dividing ISI standard deviation by mean ISI. A CV greater than 1 is an indicator of neuronal bursting and a CV equal to 1 indicates a poisson process. Repeated measures ANOVAs were performed for all three variables: CV, ISI STD and mean ISI. For CV, there was no significant main effect of THC administration [*F*_(1, 196)_ = 0.68, *p* = 0.4096], nor was there a significant interaction between drug condition and recording phase [*F*_(2, 369)_ = 1.61, *p* = 0.2008]. A significant main effect of recording phase was found [*F*_(2, 379)_ = 3.76, *p* = 0.0241] and *post-hoc* assessment showed that CVs were higher during the pre-task recording phase compared to the task phase (Figure 8A). For ISI standard deviation, neither main effect was significant [drug condition: *F*_(1, 196)_ = 2.72, *p* = 0.1004; recording phase: *F*_(2, 379)_ = 0.1, *p* = 0.9017], but the interaction between drug condition and recording phase was significant [*F*_(2, 369)_ = 3.72, *p* = 0.0252]. *Post-hoc* assessment of ISI STD revealed only two significant differences: amount of variability is greater during the task compared to either before or after in the vehicle condition (Figure 8B). For mean ISI, neither main effect was significant [drug condition: *F*_(1, 196)_ = 3.84, *p* = 0.0514; recording phase: *F*_(2, 379)_ = 1.82, *p* = 0.1633], but there was a significant interaction [*F*_(2, 369)_ = 7.2, *p* = 0.0009]. *Post-hoc* assessment for mean ISI revealed three significant differences: neurons fire slower after THC compared to vehicle during post-task recordings, and neurons fire slower during the task compared to pre- and post-task after vehicle administration (Figure 8C). Both the Hurst exponent and multifractal complexity (width) appear to be better indicators of hippocampal microcircuit processing than these standard variability measures since they were able to distinguish task phase and drug condition combinations.

### Frequency content varies in a task-specific manner

Independent delta and theta rhythms were found in the human medial temporal lobe and are hypothesized to perform separate cognitive functions (Mormann et al., 2008). Delta power is a classical marker used to assess physiological/sleep state (Harmony et al., 1996; Pereda et al., 1999; Clemens et al., 2013) and is negatively correlated with human working memory performance (Axmacher et al., 2010). Additionally, theta frequency activity is known to coordinate neuronal interactions in working memory networks (Jones and Wilson, 2005), has been correlated with performance during the DNMS task (Hyman et al., 2010, 2011) and is reduced by cannabinoid administration (Ilan et al., 2004; Robbe et al., 2006; Böcker et al., 2010; Kucewicz et al., 2011). Therefore, we assessed the performance of these frequency components in distinguishing between recording phases and drug conditions. Delta and theta power in all hippocampal neurons were assessed by calculating the total power in the delta (0.5–4 Hz) and theta bands (4–8 Hz) using a fast Fourier transform (Figures 4B,D). These two activity bands were chosen because of their prominence in hippocampal spike train recordings. Two repeated measures ANOVAs were performed using delta and theta power as the dependent variable to determine if alterations in frequency content can account for the same differences detected with multifractal analysis.

When using delta power as the dependent variable, a significant main effect of different recording phases was found [*F*_(2, 379)_ = 5.23, *p* = 0.0057]. *Post-hoc* analysis of the recording phase effect showed that delta power is greater during task and post-task compared to pre-task (Figure 8D). There was no significant main effect of drug condition [*F*_(1, 196)_ = 2.19, *p* = 0.141] or interaction between drug condition and recording phase [*F*_(2, 369)_= 2.52, *p* = 0.082].

Theta power was assessed in the same manner. The overall effect of drug condition was not significant [*F*_(1, 196)_ = 3.1, *p* = 0.0796]. However, there was a significant main effect of recording phase [*F*_(2, 379)_ = 41.91, *p* < 0.0001] and a significant drug condition by recording phase interaction [*F*_(2, 369)_ = 13.28, *p* < 0.0001]. *Post-hoc* analysis of the interaction revealed that theta power was significantly higher during both the pre- and post-task recording sessions compared to the task (Figure 8E).

### Correlation analysis

The aforementioned results suggest a relationship between spike train temporal coding properties quantified by fractality (Hurst exponent/LRTCs/self-similarity), multifractality (singularity spectrum width), variability measures (mean ISI, ISI STD, CV), and frequency content variables (delta and theta). Correlation analyses were performed in order to improve theoretical understandings of multifractal variables and provide insight for developing computational models of the nervous system that reproduce LRTCs and multifractal complexity of neuronal spike trains. Spearman's rho values were computed for all recording phases and drug condition combinations separately. Correlations between the Hurst exponent and singularity spectrum width yielded small positive rho values ranging from 0.10 to 0.28 (data not shown). This indicates that the Hurst exponent and multifractal width quantify different spike train properties with respect to hippocampal microcircuit processing. Fifty additional correlation analyses were performed to determine relationships of LRTCs (the Hurst exponent) and magnitude of multifractality (width) with CV, ISI STD, mean ISI, delta, and theta power for each recording phase and drug condition. Spearman's rho values greater than |0.5| were considered to indicate “strong” correlations. Pre-task THC values were not reported because they were similar to pre-task vehicle values in all cases.

**Figure 9:**
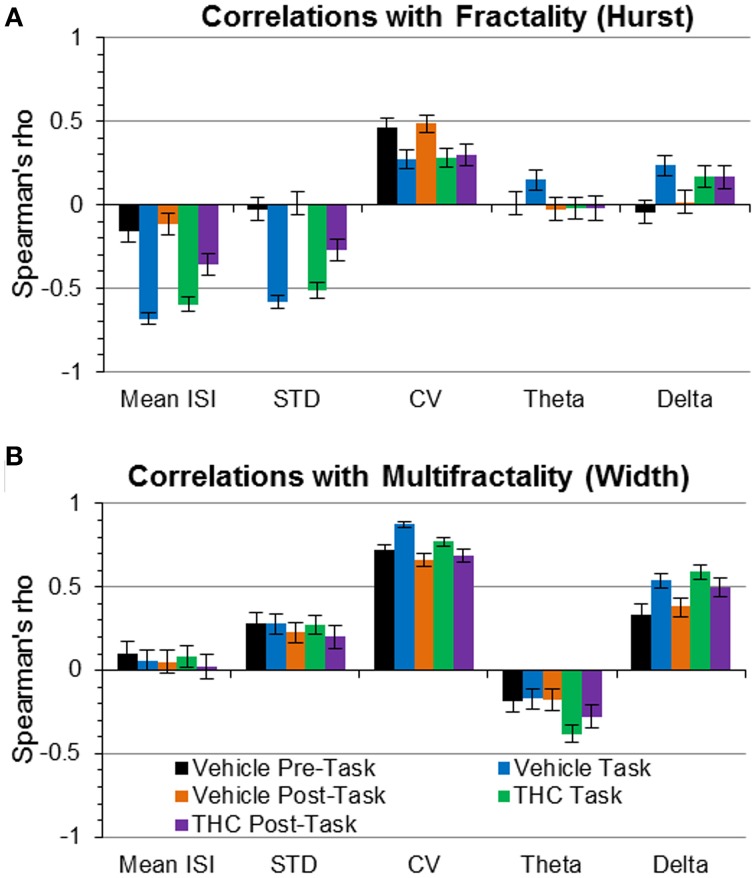
**Correlations of fractality (Hurst exponent) and multifractality (singularity spectrum width) with spike train variability and frequency spectrum measures**. Correlations (Spearman's rho) are plotted with 95% confidence intervals as error bars. **(A)** The Hurst exponent is negatively correlated with mean ISI and ISI STD during task recordings independent of drug condition—indicating that faster spiking and less variable firing patterns are correlated with greater LRTCs during the task only. **(B)** The coefficient of variation (CV) was positively correlated with multifractal complexity (width)—indicating “burstiness” correlates with multifractality. Delta power was positively correlated with multifractality (width) during the task phase, regardless of drug condition.

We tested whether LRTCs were associated with changes in variability or frequency content using correlation. A strong relationship was found between the Hurst exponent and mean ISI during task recordings (Figure 9A), indicating that LRTCs are more prominent with shorter average ISIs (i.e., when neurons fire more frequently). The Hurst exponent was negatively correlated with ISI STD during the task (Figure 9A), indicating that LRTCs and self-similarity occur more frequently when neurons fire with less variable ISIs. Both of these correlations were weaker during the resting phase recordings (pre- and post-task), suggesting a task-specific relationship of fractal vs. firing rate and variability patterns. None of the other three relationships were strong, which indicate that LRTCs and self-similarity quantified by the Hurst exponent are independent from CV, theta and delta power.

To develop a deeper conceptual understanding of multifractal complexity, the singularity spectrum width was correlated with standard variability measures and frequency content. Multifractal complexity (width) was strongly correlated with relative variability, expressed as the coefficient of variation (CV; Figure 9B), during all recording phases and drug conditions. Despite this strong correlation, multifractal complexity was able to distinguish between recording phases and drug conditions better than CV (Figure 7F vs. Figure 8B). This indicates that multifractal complexity is a more sensitive measure for detecting memory-specific alterations in hippocampal microcircuit processing. A strong positive correlation was found between multifractal complexity and delta power during the task that decreased during the resting state recordings (Figure 9B), suggesting that low frequency activity may support multifractal complexity during working memory. Multifractal complexity strongly correlated with “Poison-ness” (CV) and delta power but not with mean ISI, ISI STD, or theta power. Taken together, these results suggest that fractality, multifractality, and theta frequency activity quantify different properties of spike train temporal coding structure.

## Discussion

Multifractal analysis revealed something never shown before: the nature of the temporal structure in hippocampal spike trains is altered by performing a working memory task. Monofractality (Hurst exponent) decreased but multifractality (width) increased from pre-task to task and these trends reversed from task to post-task. Prior indications that the neuronal encoding during the task is essential for successful performance (Hampson et al., 2012) support the hypothesis that spike trains represent information with multifractal temporal coding properties (Fetterhoff et al., 2015). Previous results showed that microcircuit activity patterns occurring when encoding information during the sample phase are correlated with correct performance (Berger et al., 2011; Opris et al., 2012) and support the notion that memory functions through repetitive neuronal ensemble codes (Deadwyler and Hampson, 1997; Berger et al., 2012). Therefore, multifractal, self-similar spiking patterns detected during this study might constitute a substrate for memory information transmission.

The main goal of this study was to elucidate the multifractal properties of active hippocampal microcircuit processing by assessing differences between resting state and working memory conditions. The presented results show that multifractal complexity (indicated by singularity spectrum width) permits distinction between all recording phases and support the use of multifractal analysis in extracting variables related to cognitive state better than other commonly applied methods. Our first hypothesis was verified by the finding that long range temporal correlations (LRTCs), indicated by the Hurst exponent, were decreased during task performance compared to the resting state (Figures 6A–C,F). We verified our second hypothesis that multifractal complexity (width) of hippocampal ISI sequences is greater during active memory processing (task recording phase) compared to the resting state (pre- and post-task; Figures 7A–C,F). Unexpectedly, we found that pre-task was more multifractal than post-task (Figure 7C). We believe this could be due to a “priming” effect because the rats are more motivated for water and their hippocampal ensembles are preparing to perform the DNMS task by some form of mental rehearsal. Due to the rats' experience with the habitual daily testing procedure, they may learn to associate pre-task recording conditions with subsequent task recordings when reward is available. The rats are also more satiated during the post-task recording after receiving water during the task and this may reduce the hippocampal inputs responsible for generating multifractality during the task. This finding highlights the advantage of using multifractal analysis for detecting even subtle differences in cognitive states. These results are consistent with other findings showing that task performance elicits multifractal fluctuations restricted to task-related regions while LRTCs decrease in all analyzed brain regions, independent of task involvment (Ciuciu et al., 2012). The structure of variability, detected as multifractal complexity, becomes more intricate when hippocampal microcircuits exhibit a wider range of dynamics required for memory processing (during the task) and this alteration may elicit breakdowns of LRTCs (Figures 6F, 7F). These results suggest that enhancement of the theoretical and conceptual foundations of neuroscience is possible by applying multifractal analysis in order to achieve the larger goal of producing a dynamic portrait of the functioning brain.

THC impairs hippocampal information transmission by disrupting the multifractal spike train patterns that may constitute a basis of memory processing. THC administration reduced multifractal complexity during the DNMS task and decreased LRTCs during post-task recording phase (Figures 6F, 7F). Under control conditions, multifractal complexity (width) and LRTCs became lower and higher, respectively, when rats transitioned from the task to the post-task, but THC administration inhibited these changes. Therefore, it is possible that THC effectively prevented endogenous microcircuit dynamics that facilitate neurophysiological adjustments from memory processing to a resting state. These findings are consistent with others demonstrating that THC promotes default mode network activity at inappropriate times (Bossong et al., 2013), and they support the application of multifractal complexity as a marker of network involvment if the reduction of multifractal complexity is due to decreased memory-related interactions that occur when DNMS performance is impaired by THC.

Computational models attempting to reproduce the temporal coding properties of neuronal spike trains (Goris et al., 2014) can likely be improved by replicating endogenously occuring LRTCs (Fürstenau, 2010) and multifractal complexity. Interpretation of the structure of variability assessed using multifractal analysis was enhanced by comparing performance and relationships between monofractal and multifractal variables (Hurst and width) and standard measures of variability. The correlation analysis showed that the Hurst exponent is negatively correlated with mean ISI and ISI STD during the DNMS task under both vehicle and THC conditions (Figure 9A). This task-specific correlation demonstrates that the increase of LRTCs during resting phase (pre/post) recordings (Figure 6F) is not due to independent changes in mean ISI and ISI STD but may be related to alterations in their ratio when expressed as CV (Figure 9A). The strongest relationship was found between multifractal complexity (width) and the coefficient of variation (Figure 9B), and this finding would suggest that multifractal complexity reflects information directly related to how ISI variability scales with ISI mean. However, the superiority of singularity spectrum width in distinguishing between examined cognitive states is clear in the population ANOVA results: multifractality (width) confirmed four out of six *ad hoc* distinctions from the interaction between drug condition and recording phase while CV failed to yield a statistically significant interaction and could only distinguish between the pre-task and task recording phases (Figure 7F vs. Figure 8A).

Examination of brain oscillations via frequency content is a commonly used method to study temporal information processing in the nervous system (Lisman, 2005; Axmacher et al., 2006; Dragoi and Buzsáki, 2006). Therefore, it is important to understand how these methods compare to new ways of quantifying and describing temporal coding properties, such as multifractal analysis. It was found that the combination of delta and theta power quantification distinguished between recording phases to a lesser extent than multifractal complexity (width) and LRTCs (Hurst). We found that theta power determination permitted distinction between two out of six *ad hoc* differences (Figure 8E), and interestingly, both were identical to changes detected by the Hurst exponent (Figure 6F). Further inspection using correlation analyses promoted discovery that the Hurst exponent was not correlated with oscillatory activity in the tested frequency bands and therefore suggests that they are independent dynamical processes. Theta and delta power were negatively and positively, respectively, correlated with multifractal complexity (width). This supports the finding that both delta and theta rhythms exist independently in the human hippocampus (Mormann et al., 2008) and suggests that temporal coding properties detected as multifractality may preferentially arise from the delta frequency activity. It is possible that the precise action potential timing in the delta frequency range, from 1 s to 250 ms, conveys essential information utilized to support cognitive function and reflected as multifractality. It is shown here that multifractal analysis can be used to detect changes in hippocampal processing better than standard Fourier spectrum analyses. However, it will be necessary to compare both methods in order to integrate multifractal concepts with prior findings describing frequency content and to ultimately synthesize new hypotheses about how the brain functions.

**Figure 10:**
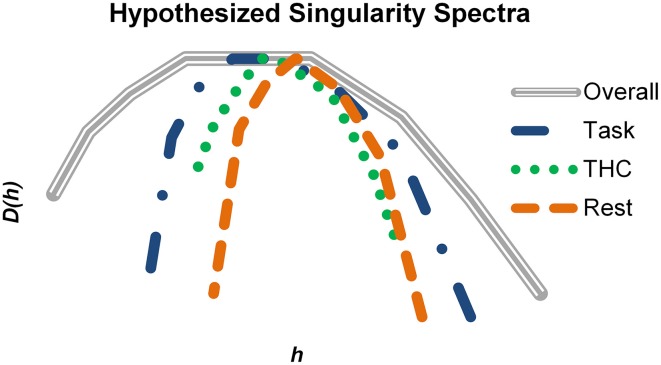
**Summary of how multifractal complexity relates to memory processing**. Hypothetical singularity spectra were constructed based on the qualitative results of the study. The gray spectrum represents the maximal amount of multifractal complexity possible in a system (i.e., hippocampal spike trains in this example). Active memory processing recruits a large portion of this potential, but additional resources are still available to support more cognitively demanding instances. THC impaired working memory and reduced multifractal complexity. During resting conditions, the singularity spectrum shrinks compared to the task (blue) condition. Multifractality may be highest when embedded information relevant for working memory is being processed. Consequently, resting and pharmacological impairment could reduce multifractal complexity by decreasing the fraction of utilized resources.

Learning and memory require information to be carried from the past into the future, and multifractal complexity of hippocampal neurons may fluctuate depending on how strongly information is received, processed and sent by these neurons. To put our results into a more general perspective, hypothetical singularity spectra were constructed to match our qualitative findings with respect to memory processing (Figure 10). We hypothesize that physiological states are characterized by specific monofractal and multifractal features. All different states must fall within a range of possible dynamics (Figure 10, gray spectrum) and thus a range of possible multifractality. Active memory processing (Figure 10, blue) recruits this system to a stronger degree than resting (Figure 10, orange) and therefore neurons recorded during the task exhibit stronger multifractality. This multifractality may facilitate memory processing by offering a larger range of spike train variability and greater processing capacity. When THC or other memory impairing agents are administered during the task (Figure 10, green), the normal level of multifractal complexity exhibited is reduced and memory performance suffers. Alterations in multifractal complexity may reflect the degree of presently embedded information and therefore would provide information relevant for detection of physiological state.

The application of multifractal analysis revealed that repeating spatiotemporal activity patterns detected in hippocampal spike trains may form a previously undiscovered contribution to the temporal coding of memory information. This demonstrated how analysis of the multifractal structure of temporal dynamics can enable new insights about how the brain functions and can facilitate methodological improvements for detection of alterations in cognitive, physiological, and pathological states (Suckling et al., 2008; Wink et al., 2008). Multifractal analysis is being utilized in pathology diagnosis and has already been successfully applied to Parkinson's Disease (Zheng et al., 2005), seizure detection (Dutta et al., 2014), Alzheimer's Disease (Vysata et al., 2013), and many others (Slezin et al., 2007; Di Ieva et al., 2015). As for physiological state, multifractal analysis was proposed as a method for automatic detection of sleep stages (Weiss et al., 2009; Zorick and Mandelkern, 2013), and an fMRI study showed changes in multifractality selectively occur in task-related brain regions (Ciuciu et al., 2012). The presented assessment of cognitive state can be integrated with these results concerning physiological state: deep sleep contains more LRTCs and less multifractality compared to REM sleep (Weiss et al., 2009), REM sleep contains larger LRTCs than waking (Zorick and Mandelkern, 2013), and as shown here, hippocampal neurons during a task-independent (resting) state exhibit greater LRTCs but less multifractality compared to when recorded during the working memory DNMS task. It is therefore possible that consciousness, described in terms of cognitive/physiological state, occurs on a spectrum that can be quantified and understood via computation of the multifractal singularity spectrum. Multifractal analysis quantifies the scale invariant, self-similar structure that is pervasive throughout biological processes and application of this method to neurophysiological data will improve our understanding of how the nervous system processes cognitive information.

## Author contributions

DF, RK, IO, CS, VM, SD, and RH designed research. DF performed the experiments. DF, RK, and RS analyzed data. All authors discussed results and commented on the paper.

### Conflict of interest statement

The authors declare that the research was conducted in the absence of any commercial or financial relationships that could be construed as a potential conflict of interest.

## References

[B1] Assenza G. Pellegrino G. Tombini M. Di Pino G. Tomasevic L. Tecchio F. (2013). Delta waves increase after cortical plasticity induction during wakefulness. Clin. Neurophysiol. 124, 1221–1227. 10.1016/j.clinph.2014.09.029 25631611

[B2] Axmacher N. Lenz S. Haupt S. Elger C. E. Fell J. (2010). Electrophysiological signature of working and long-term memory interaction in the human hippocampus. Eur. J. Neurosci. 31, 177–188. 10.1111/j.1460-9568.2009.07041.x 20092564

[B3] Axmacher N. Mormann F. Fernández G. Elger C. E. Fell J. (2006). Memory formation by neuronal synchronization. Brain Res. Rev. 52, 170–182. 10.1016/j.brainresrev.2006.01.007 16545463

[B4] Berger T. W. Hampson R. E. Song D. Goonawardena A. Marmarelis V. Z. Deadwyler S. A. (2011). A cortical neural prosthesis for restoring and enhancing memory. J. Neural Eng. 8:46017 10.1088/1741-2560/8/4/046017 PMC3141091 21677369

[B5] Berger T. W. Song D. Chan R. H. Marmarelis V. Z. LaCoss J. Wills J.. (2012). A hippocampal cognitive prosthesis: multi-input, multi-output nonlinear modeling and VLSI implementation. IEEE Trans. Neural Syst. Rehabil. Eng. 20, 198–211. 10.1109/tnsre.2012.2189133 22438335 PMC3395724

[B6] Böcker K. B. E. Hunault C. C. Gerritsen J. Kruidenier M. Mensinga T. T. Kenemans J. L. (2010). Cannabinoid modulations of resting state EEG θ power and working memory are correlated in humans. J. Cogn. Neurosci. 22, 1906–1916. 10.1162/jocn.2009.21355 19803687

[B7] Bossong M. G. Jansma J. M. van Hell H. H. Jager G. Kahn R. S. Ramsey N. F. (2013). Default mode network in the effects of Δ9-Tetrahydrocannabinol (THC) on human executive function. PLoS ONE 8:e70074. 10.1371/journal.pone.0070074 23936144 PMC3729458

[B8] Buzsáki G. Moser E. I. (2013). Memory, navigation and theta rhythm in the hippocampal-entorhinal system. Nat. Neurosci. 16, 130–138. 10.1038/nn.3304 23354386 PMC4079500

[B9] Ciuciu P. Varoquaux G. Abry P. Sadaghiani S. Kleinschmidt A. (2012). Scale-Free and multifractal time dynamics of fMRI signals during rest and task. Front. Physiol. 3:186 10.3389/fphys.2012.00186 PMC3375626 22715328

[B10] Clemens Z. Borbély C. Weiss B. Eross L. Szucs A. Kelemen A.. (2013). Increased mesiotemporal delta activity characterizes virtual navigation in humans. Neurosci. Res. 76, 67–75. 10.1016/j.neures.2013.03.004 23524244

[B11] Deadwyler S. A. Bunn T. Hampson R. E. (1996). Hippocampal ensemble activity during spatial delayed-nonmatch-to-sample performance in rats. J. Neurosci. 16, 354–372. 8613802 10.1523/JNEUROSCI.16-01-00354.1996 PMC6578714

[B12] Deadwyler S. A. Hampson R. E. (1997). The significance of neural ensemble codes during behavior and cognition. Annu. Rev. Neurosci. 20, 217–244. 10.1146/annurev.neuro.20.1.217 9056713

[B13] De Carli F. Nobili L. Beelke M. Watanabe T. Smerieri A. Parrino L.. (2004). Quantitative analysis of sleep EEG microstructure in the time-frequency domain. Brain Res. Bull. 63, 399–405. 10.1016/j.brainresbull.2003.12.013 15245767

[B14] Di Ieva A. Esteban F. J. Grizzi F. Klonowski W. Martín-Landrove M. (2015). Fractals in the neurosciences, part II: clinical applications and future perspectives. Neuroscientist 21, 30–43. 10.1177/1073858413513928 24362814

[B15] Dixon J. A. Holden J. G. Mirman D. Stephen D. G. (2012). Multifractal dynamics in the emergence of cognitive structure. Top. Cogn. Sci. 4, 51–62. 10.1111/j.1756-8765.2011.01162.x 22253177

[B16] Dragoi G. Buzsáki G. (2006). Temporal encoding of place sequences by hippocampal cell assemblies. Neuron 50, 145–157. 10.1016/j.neuron.2006.02.023 16600862

[B17] Dutta S. Ghosh D. Samanta S. Dey S. (2014). Multifractal parameters as an indication of different physiological and pathological states of the human brain. Phys. A Stat. Mech. Appl. 396, 155–163. 10.1016/j.physa.2013.11.014

[B18] Fetterhoff D. Opris I. Simpson S. L. Deadwyler S. A. Hampson R. E. Kraft R. A. (2015). Multifractal analysis of information processing in hippocampal neural ensembles during working memory under Δ9-tetrahydrocannabinol administration. J. Neurosci. Methods 244, 136–153. 10.1016/j.jneumeth.2014.07.013 25086297 PMC4312266

[B19] Fürstenau N. (2010). A nonlinear dynamics model for simulating long range correlations of cognitive bistability. Biol. Cybern. 103, 175–198. 10.1007/s00422-010-0388-4 20405140

[B20] Gaoni Y. Mechoulam R. (1964). Isolation, structure, and partial synthesis of an active constituent of hashish. J. Am. Chem. Soc. 86, 1646–1647. 10.1021/ja01062a046

[B21] Garn H. Waser M. Deistler M. Schmidt R. Dal-Bianco P. Ransmayr G.. (2014). Quantitative EEG in Alzheimer's disease: cognitive state, resting state and association with disease severity. Int. J. Psychophysiol. 93, 390–397. 10.1016/j.ijpsycho.2014.06.003 24933410

[B22] Goris R. L. T. Movshon J. A. Simoncelli E. P. (2014). Partitioning neuronal variability. Nat. Neurosci. 17, 858–865. 10.1038/nn.3711 24777419 PMC4135707

[B23] Hampson R. E. Deadwyler S. A. (1999). Cannabinoids, hippocampal function and memory. Life Sci. 65, 715–723. 10.1016/S0024-3205(99)00294-5 10462072

[B24] Hampson R. E. Deadwyler S. A. (2000). Cannabinoids reveal the necessity of hippocampal neural encoding for short-term memory in rats. J. Neurosci. 20, 8932–8942. 11102504 10.1523/JNEUROSCI.20-23-08932.2000 PMC6773063

[B25] Hampson R. E. Simeral J. D. Deadwyler S. A. (1999). Distribution of spatial and nonspatial information in dorsal hippocampus. Nature 402, 610–614. 10.1038/45154 10604466

[B26] Hampson R. E. Simeral J. D. Kelly E. J. Deadwyler S. A. (2003). Tolerance to the memory disruptive effects of cannabinoids involves adaptation by hippocampal neurons. Hippocampus 13, 543–556. 10.1002/hipo.10081 12921345

[B27] Hampson R. E. Song D. Chan R. H. Sweatt A. J. Riley M. R. Goonawardena A. V.. (2012). Closing the loop for memory prosthesis: detecting the role of hippocampal neural ensembles using nonlinear models. IEEE Trans. Neural Syst. Rehabil. Eng. 20, 510–525. 10.1109/tnsre.2012.2190942 22498704 PMC3395725

[B28] Harmony T. Fernández T. Silva J. Bernal J. Díaz-Comas L. Reyes A.. (1996). EEG delta activity: an indicator of attention to internal processing during performance of mental tasks. Int. J. Psychophysiol. 24, 161–171. 10.1016/S0167-8760(96)00053-0 8978441

[B29] Hasselmo M. E. Stern C. E. (2014). Theta rhythm and the encoding and retrieval of space and time. Neuroimage 85(Pt 2), 656–666. 10.1016/j.neuroimage.2013.06.022 23774394 PMC3918488

[B30] Hurst H. E. (1951). Long-term storage capacity of reservoirs. Trans. Am. Soc. Eng. 116, 770–808.

[B31] Hyman J. M. Hasselmo M. E. Seamans J. K. (2011). What is the functional relevance of prefrontal cortex entrainment to hippocampal theta rhythms? Front. Neurosci. 5:24 10.3389/fnins.2011.00024 PMC3052540 21427795

[B32] Hyman J. M. Zilli E. A. Paley A. M. Hasselmo M. E. (2010). Working memory performance correlates with prefrontal-hippocampal theta interactions but not with prefrontal neuron firing rates. Front. Integr. Neurosci. 4:2. 10.3389/neuro.07.002.2010 20431726 PMC2861479

[B33] Ihlen E. A. F. (2012). Introduction to multifractal detrended fluctuation analysis in matlab. Front. Physiol. 3:141 10.3389/fphys.2012.00141 PMC3366552 22675302

[B34] Ihlen E. A. F. Vereijken B. (2010). Interaction-dominant dynamics in human cognition: beyond 1/f(alpha) fluctuation. J. Exp. Psychol. Gen. 139, 436–463. 10.1037/a0019098 20677894

[B35] Ilan A. B. Smith M. E. Gevins A. (2004). Effects of marijuana on neurophysiological signals of working and episodic memory. Psychopharmacology (Berl.) 176, 214–222. 10.1007/s00213-004-1868-9 15502936 PMC1463999

[B36] Ivanov P. C. Amaral L. A. Goldberger A. L. Havlin S. Rosenblum M. G. Struzik Z. R.. (1999). Multifractality in human heartbeat dynamics. Nature 399, 461–465. 10.1038/20924 10365957

[B37] Jones M. W. Wilson M. A. (2005). Theta rhythms coordinate hippocampal-prefrontal interactions in a spatial memory task. PLoS Biol 3:e402. 10.1371/journal.pbio.0030402 16279838 PMC1283536

[B38] Kantelhardt J. W. (2012). Fractal and multifractal time series, in Mathematics of Complexity and Dynamical Systems, ed Meyers R. A. (New York, NY: Springer), 463–487.

[B39] Kantelhardt J. W. Zschiegner S. A. Koscielny-Bunde E. Havlin S. Bunde A. Stanley H. E. (2002). Multifractal detrended fluctuation analysis of nonstationary time series. Phys. A. Stat. Mech. Appl. 316, 87–114. 10.1016/S0378-4371(02)01383-3

[B40] Kelty-Stephen D. G. Palatinus K. Saltzman E. Dixon J. A. (2013). A tutorial on multifractality, cascades, and interactivity for empirical time series in ecological science. Ecol. Psychol. 25, 1–62. 10.1080/10407413.2013.753804

[B41] Kucewicz M. T. Tricklebank M. D. Bogacz R. Jones M. W. (2011). Dysfunctional prefrontal cortical network activity and interactions following cannabinoid receptor activation. J. Neurosci. 31, 15560–15568. 10.1523/JNEUROSCI.2970-11.2011 22031901 PMC6703515

[B42] Lahmiri S. Boukadoum M. (2013). Alzheimer's Disease detection in brain magnetic resonance images using multiscale fractal analysis. ISRN Radiol. 2013, 1–7. 10.5402/2013/627303 PMC4045563 24967286

[B43] Linkenkaer-Hansen K. Nikouline V. V. Palva J. M. Ilmoniemi R. J. (2001). Long-range temporal correlations and scaling behavior in human brain oscillations. J. Neurosci. 21, 1370–1377. 11160408 10.1523/JNEUROSCI.21-04-01370.2001 PMC6762238

[B44] Lisman J. (2005). The theta/gamma discrete phase code occuring during the hippocampal phase precession may be a more general brain coding scheme. Hippocampus 15, 913–922. 10.1002/hipo.20121 16161035

[B45] Mormann F. Osterhage H. Andrzejak R. G. Weber B. Fernández G. Fell J.. (2008). Independent delta/theta rhythms in the human hippocampus and entorhinal cortex. Front. Hum. Neurosci. 2:3. 10.3389/neuro.09.003.2008 18958204 PMC2525973

[B46] Nguyen D. P. Barbieri R. Wilson M. A. Brown E. N. (2008). Instantaneous frequency and amplitude modulation of EEG in the hippocampus reveals state dependent temporal structure. Conf. Proc. IEEE Eng. Med. Biol. Soc. 2008, 1711–1715. 10.1109/IEMBS.2008.4649506 19163009

[B47] Opris I. Hampson R. E. Gerhardt G. A. Berger T. W. Deadwyler S. A. (2012). Columnar processing in primate prefrontal cortex: evidence for executive control microcircuits. J. Cogn. Neurosci. 24, 2334–2347. 10.1162/jocn_a_00307 23016850 PMC3754813

[B48] Palva S. Monto S. Palva J. M. (2010). Graph properties of synchronized cortical networks during visual working memory maintenance. Neuroimage 49, 3257–3268. 10.1016/j.neuroimage.2009.11.031 19932756

[B49] Paxinos G. Watson C. (1997). The Rat Brain in Stereotaxic Coordinates. San Diego, CA: Academic Press.

[B50] Pereda E. Gamundi A. Nicolau M. C. Rial R. González J. (1999). Interhemispheric differences in awake and sleep human EEG: a comparison between non-linear and spectral measures. Neurosci. Lett. 263, 37–40. 10.1016/S0304-3940(99)00104-4 10218905

[B51] Robbe D. Montgomery S. M. Thome A. Rueda-Orozco P. E. McNaughton B. L. Buzsaki G. (2006). Cannabinoids reveal importance of spike timing coordination in hippocampal function. Nat. Neurosci. 9, 1526–1533. 10.1038/nn1801 17115043

[B52] Sato N. Yamaguchi Y. (2003). Memory encoding by theta phase precession in the hippocampal network. Neural Comput. 15, 2379–2397. 10.1162/089976603322362400 14511526

[B53] Serletis D. Bardakjian B. L. Valiante T. A. Carlen P. L. (2012). Complexity and multifractality of neuronal noise in mouse and human hippocampal epileptiform dynamics. J. Neural Eng. 9:056008. 10.1088/1741-2560/9/5/056008 22929878

[B54] Slezin V. B. Korsakova E. A. Dytjatkovsky M. A. Schultz E. A. Arystova T. A. Siivola J. R. (2007). Multifractal analysis as an aid in the diagnostics of mental disorders. Nord. J. Psychiatry 61, 339–342. 10.1080/08039480701643175 17990194

[B55] Suckling J. Wink A. M. Bernard F. A. Barnes A. Bullmore E. (2008). Endogenous multifractal brain dynamics are modulated by age, cholinergic blockade and cognitive performance. J. Neurosci. Methods 174, 292–300. 10.1016/j.jneumeth.2008.06.037 18703089 PMC2590659

[B56] Van Someren E. J. W. Van Der Werf Y. D. Roelfsema P. R. Mansvelder H. D. da Silva F. H. L. (2011). Slow brain oscillations of sleep, resting state, and vigilance. Prog. Brain Res. 193, 3–15. 10.1016/B978-0-444-53839-0.00001-6 21854952

[B57] Vysata O. Prochazka A. Mare J. Rusina R. Pazdera L. Vali M.. (2013). Change in the characteristics of EEG color noise in Alzheimer's Disease. Clin. EEG Neurosci. 45, 147–151. 10.1177/1550059413491558 24131619

[B58] Weiss B. Clemens Z. Bódizs R. Vágó Z. Halász P. (2009). Spatio-temporal analysis of monofractal and multifractal properties of the human sleep EEG. J. Neurosci. Methods 185, 116–124. 10.1016/j.jneumeth.2009.07.027 19646476

[B59] Wink A.-M. Bullmore E. Barnes A. Bernard F. Suckling J. (2008). Monofractal and multifractal dynamics of low frequency endogenous brain oscillations in functional MRI. Hum. Brain Mapp. 29, 791–801. 10.1002/hbm.20593 18465788 PMC6870616

[B60] Zheng Y. Gao J. Sanchez J. C. Principe J. C. Okun M. S. (2005). Multiplicative multifractal modeling and discrimination of human neuronal activity. Phys. Lett. A 344, 253–264. 10.1016/j.physleta.2005.06.092

[B61] Zorick T. Mandelkern M. A. (2013). Multifractal detrended fluctuation analysis of human EEG: preliminary investigation and comparison with the wavelet transform modulus maxima technique. PLoS ONE 8:e68360. 10.1371/journal.pone.0068360 23844189 PMC3700954

