# Supplementary material for: Distinguishing cognitive state with multifractal complexity of hippocampal interspike interval sequences
Source: Front Syst Neurosci. 2015 Sep 17;9:130. doi: 10.3389/fnsys.2015.00130 (PMC4585000; doi:10.3389/fnsys.2015.00130)
Supplement: Supplementary file 1 [file DataSheet1.DOCX]

**Figure S1. Waveform Identification across recording sessions.** All waveform files (.plx) were concatenated into one large file, and waveform sorting was performed only on this merged file. After sorting, the file was separated into appropriate files based on digital timestamps acquired during recording. Waveforms, PCA space and ISI histograms are shown for unsorted noise (gray) and two identified neurons (yellow and green) from one wire located in CA1. The green neuron was used as the example in figure 2. Images were taken directly from Plexon Offline Sorter. **(A)** Waveforms (left), PCA space (upper right), and ISI histograms (bottom right) from the merged file are displayed. Sorting a merged file ensures that similar waveform shaped are isolated on every session. **(B)** Waveforms, PCA space and ISI histograms are shown for the vehicle session. **(C)** Waveforms, PCA space and ISI histograms are shown for the THC session.

**Figure S2. Monofractal and Multifractal changes in Hippocampal CA3 and CA1 regions.** (A) and (C) are reproduced from figures 6F and 7F for comparison purposes. Errors bars represent S.E.M. Statistical significance is designated by * indicating p < 0.0083. **(A)** Each bar was obtained by averaging Hurst exponent values from individual spike trains within specified recording phase and drug treatment combinations (n = 771-1004 neurons per group). **(B)** Three differences were found between CA3 and CA1 for the Hurst exponent, signified by asterisks. CA3 neurons exhibited stronger monofractality during vehicle resting states (pre and post) compared to CA1. Conversly, the Hurst exponent of CA1 neurons was larger during the task. Besides that, the same 3 differences were found within each region as the population data shown in (A), but within region significance is not denoted to preserve clarity. Each bar was obtained by averaging Hurst exponent values from individual spike trains within specified recording phase and drug treatment combinations (n = 592-443 CA1 neurons and 412-315 CA3 neurons per group). **(C)** Each bar was obtained by averaging multifractality (width *h)* from individual spike trains within specified recording phase and drug treatment combinations (n = 771-1004 neurons per group). **(D)** Two between-region differences were detected and are marked by asterisks: CA3 neurons recorded during the task are less multifractal than CA1 neurons recorded during the task under both drug conditions. The within-region differences are signified on the graph using lines between groups. CA3 neurons had greater multifractality during task control recordings compared to task THC but did not present the other three population differences shown in (C). CA1 neurons had 3 of 4 detected population differences (pre-vehicle vs. task-vehicle, task-vehicle vs. post-vehicle, and task-vehicle vs. post-vehicle). Pre-control vs. post-control was not significant within CA1. Each bar was obtained by averaging multifractality values from individual spike trains within specified recording phase and drug treatment combinations (n = 592-443 CA1 neurons and 412-315 CA3 neurons per group).
