# Supplementary figures and images for: Distinguishing cognitive state with multifractal complexity of hippocampal interspike interval sequences
Source: Front Syst Neurosci. 2015 Sep 17;9:130. doi: 10.3389/fnsys.2015.00130 (PMC4585000; doi:10.3389/fnsys.2015.00130)

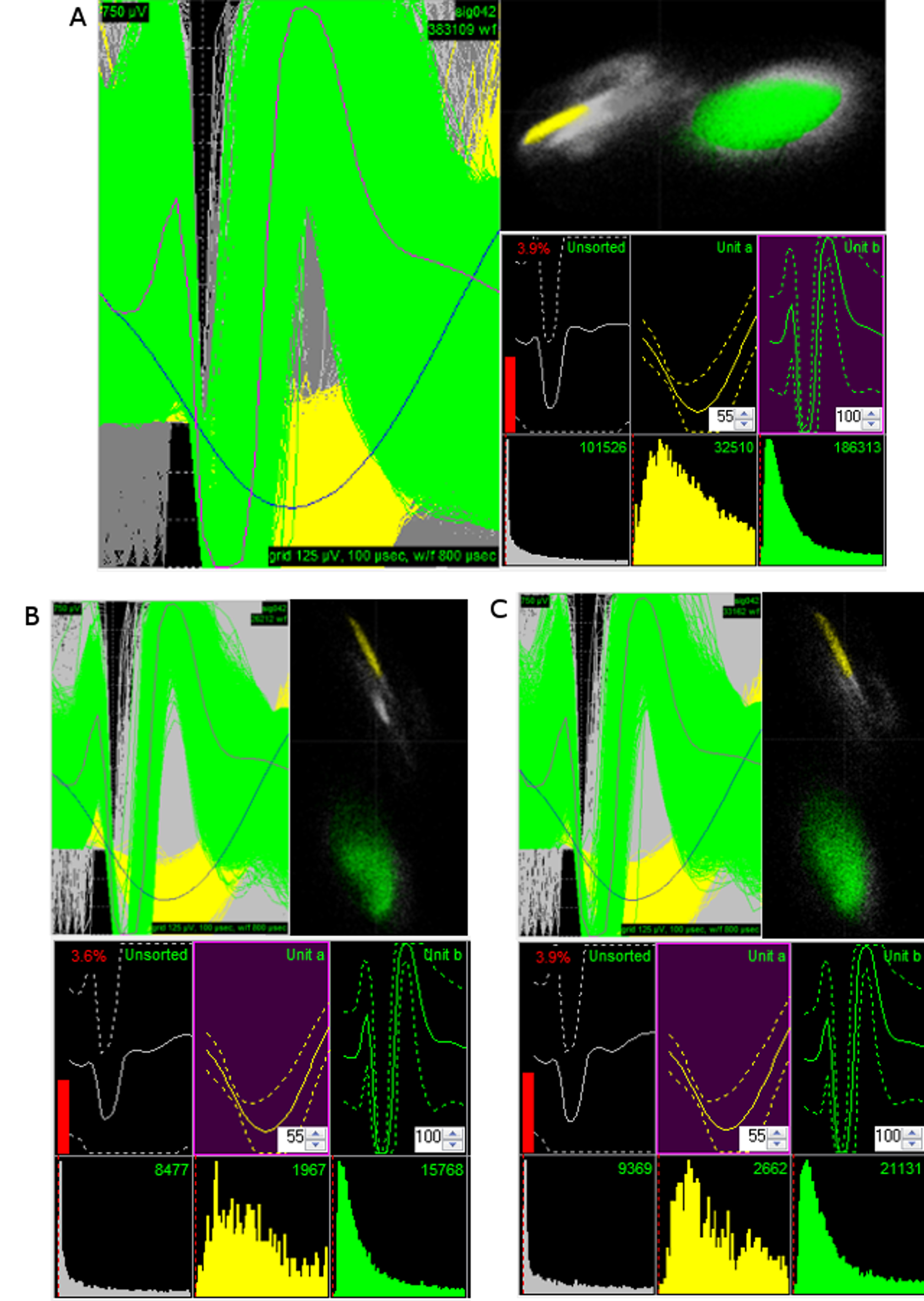

Supplement: Supplementary file 2 [file Image1.TIF]

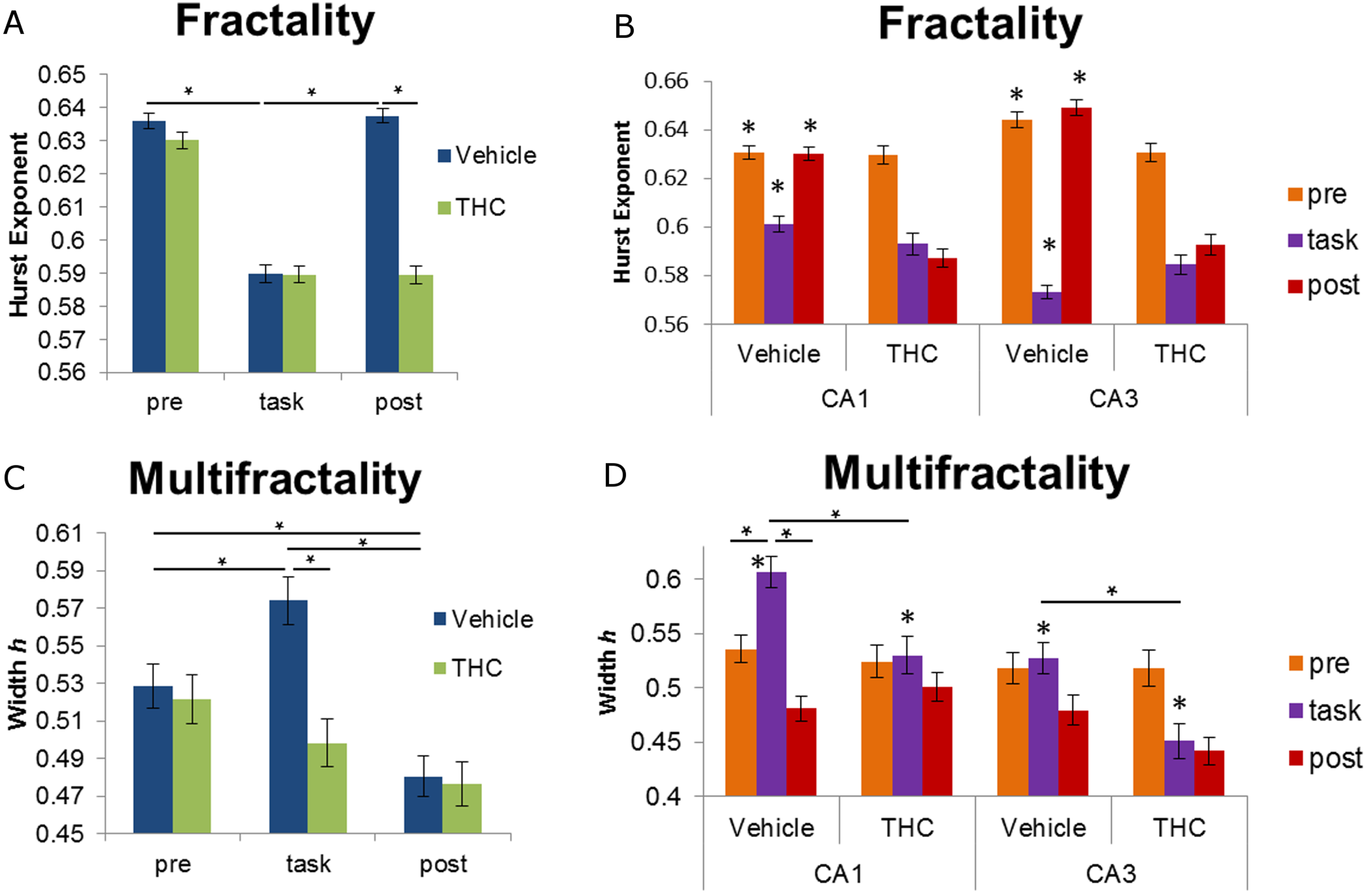

Supplement: Supplementary file 3 [file Image2.TIF]
